# Physiological Reconstruction for Moderate–Severe Pelvic Organ Prolapse: A Multicenter Retrospective Self-Controlled Study

**DOI:** 10.1007/s11596-025-00095-3

**Published:** 2025-07-30

**Authors:** Zhen-hua Gao, Xing-qi Wang, Kun-bin Ke, Quan Zhang, Ling Li, Ji-hong Shen

**Affiliations:** 1 Department of Urology, The First Affiliated Hospital of Kunming Medical University, Kunming, 650032 China; 2 Yunnan Province Clinical Research Center for Chronic Kidney Disease, Kunming, 650032 China

**Keywords:** Physiological reconstruction, Pelvic organ prolapse, Pelvic floor reconstruction, Mesh implantation, Surgical outcomes, Treatment efficacy, Complications, Retrospective analysis

## Abstract

**Objective:**

This is a self-controlled multicenter retrospective study based on the clinical efficacy and complications of physiological reconstruction in the treatment of moderate and severe pelvic organ prolapse.

**Methods:**

From December 2014 to August 2021, 517 women were included and registered for physiological reconstruction at four Chinese urogynecology institutions. We enrolled 364 women with POP-Q stage ≥ 3. The degree of POP was quantified via a POP-Q system. The surgical purpose of physiological reconstruction is to repair the vagina, levator ani muscle, perineum, and urogenital hiatus and adopt a repair method in accordance with the axial direction of physiology. All 330 evaluable participants were followed for 2 years. The evaluation indices included the PFDI-20, PGI-I, PFIQ-7, PISQ-12, PGI-I, and PGI-S. All complications were coded according to the category-time-site system proposed by the International Urogynecological Association (IUGA) and International Continence Society (ICS).

**Results:**

Compared with the preoperative POP-Q scores, statistically significant improvements were observed at the 6-month, 1-year and 2-year time points (*P* < 0.001). Statistically significant improvements in quality of life were observed across all time points.

**Conclusions:**

Physiologic reconstructive surgical techniques combined with modified anterior pelvic floor mesh implantation could help restore the physiologic axis and vaginal shape, which may be the most important factors in maintaining the functional position of pelvic floor organs and is the most effective method for repairing the pelvic fascia tendon arch. This surgical method is safe, feasible, and effective in patients with severe prolapse.

**Supplementary Information:**

The online version contains supplementary material available at 10.1007/s11596-025-00095-3.

## Introduction

As modern humans walk upright, pelvic organ prolapse (POP) is a specific human disease. The prevalence of POP is approximately 40%–60% in parous women [1, 2]. It is a mechanical imbalance caused by complex damage to various pelvic floor structures. A number of studies based on pelvic floor finite element and three-dimensional magnetic resonance reconstruction [3, 4] revealed that changes in the axial direction of pelvic floor biomechanics, such as changes in the shape and angle of the vagina, weakening of the support strength of the levator muscle group, and loss of the mechanical support plane of the pelvic septum, lead to the occurrence and development of POP. With the establishment of three-chamber and three-level theories, apical suspension has become the gold standard for the treatment of POP [5]. However, problems such as cure rates and complication rates still exist, and patients do not achieve satisfactory treatment outcomes [6]. Moreover, many researchers ignore the fact that defects of the pelvic floor caused by vaginal delivery are omnibearing, multilevel, and multistructure injuries. This may lead to POP recurrence or compartment prolapse [7].

Therefore, based on the restoration of pelvic floor physiological mechanical homeostasis, we used synthetic non-absorbable vaginal mesh (TVM) technology to restore anterior pelvic support, along with posterior colporrhaphy (rectovaginal fascial plication) and reconstruction of the perineal body (perineorrhaphy) to achieve a normal vaginal axial and pelvic septum shape. This study aimed to evaluate the success rate of physiological reconstruction for severe POP. We investigated the subjective and objective outcomes of the physiological reconstruction of POP with a minimum follow-up period of two years. Accordingly, we assessed our experience with this surgical procedure, focusing on safety, operative characteristics, and both subjective and anatomical outcomes after two years of assessment.

## Materials and Methods

### Study Design

This self-controlled multicenter retrospective study was conducted at The First Affiliated Hospital of Kunming Medical University, Gansu Provincial Hospital, The First Hospital of Shanxi Medical University, and The Affiliated Hospital of Medical School, Ningbo University in China. From December 2014 to August 2021, 517 women were included and registered for physiological reconstruction and 364 POP female patients who underwent physiological reconstruction without stress urinary incontinence surgery were retrospectively selected.

### Inclusion and Exclusion Criteria

**Fig. 1:**
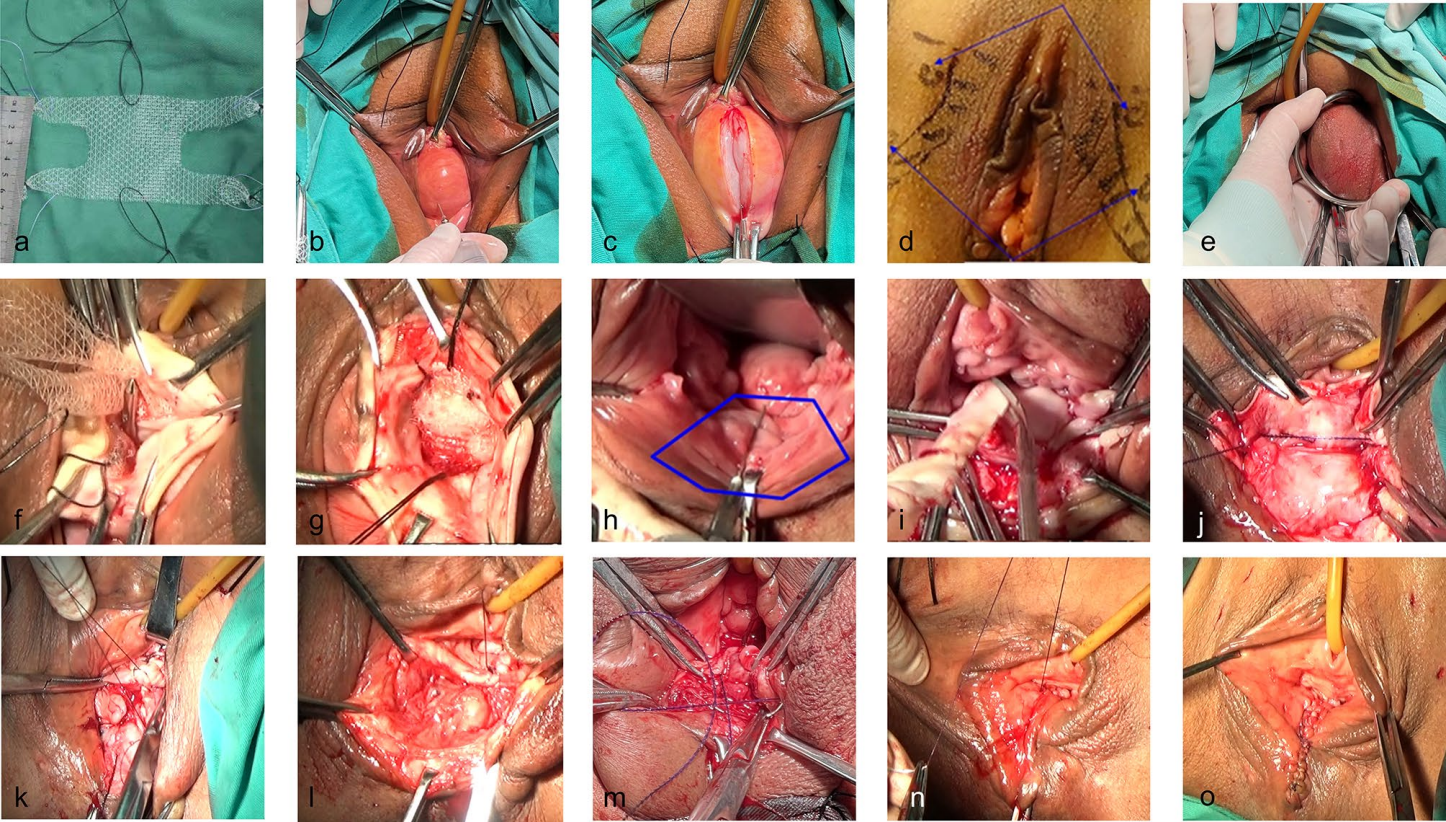
**a** A polypropylene mesh was cut to obtain four long arms (approximately 2 cm × 5 cm) and a central rectangular part (approximately 5–8 cm × 5 cm). **b** Water separation. **c** Make an incision on the anterior vaginal wall. **d** Anatomical landmark. **e, f** The mesh was placed by retracting the introducer needles. **g** Fix the mesh on the vaginal wall to prevent the mesh from shifting. **h** Make a hexagonal incision on the posterior vaginal wall. **i** The excess vaginal epithelium was trimmed. **j** Reconstruct the levator ani muscle and the genital hiatus. **k** The upper half of the posterior vaginal epithelium was closed. **l, m** Reconstruct the perineal body. **n** The lower half of the posterior vaginal epithelium was closed. **o** The mesh was tightened and cut

The degree of POP was quantified via the POP-Q system [8]. The study included all patients with POP-Q stage ≥ 3. The baseline estimation included complete history, physical examination, urodynamic test, and multiple validated questionnaires. Urodynamic tests were performed in accordance with the International Continence Society (ICS) recommendations, and occult stress urinary incontinence was assessed via pessary placement. The exclusion criterion was cervical elongation, which was defined as a cervical length > 33.8 mm [9]. Approval from the First Affiliated Hospital of Kunming Medical University institutional review board was obtained for the study protocol. Before and after surgery, local conjugated estrogen vaginal cream was used for one month to prevent mesh exposure. Ultralight macroporous mesh (Pelvimesh®; Herniamesh®, Chivasso, Italy), which is made from non-absorbable single-filament polypropylene, was used in this study. Before the mesh was implanted, it was tailored to form a rectangle-shaped central body, from which two pairs of slings branched off: upper and lower. Figure 1a shows the mesh and how it was precut.

### Preoperative and Perioperative Assessment

The validated questionnaires were used for preoperative and postoperative evaluation via the Pelvic Floor Distress Inventory-20 (PFDI-20) [10], Pelvic Floor Impact Questionnaire-7 (PFIQ-7) [10], POP/Urinary Incontinence Sexual Questionnaire short form (PISQ-12) [11], Patient Global Impression of Improvement (PGI-I), and Patient Global Impression of Severity (PGI-S) [12].

Outcomes were assessed via an abbreviated history, pelvic examination with POP-Q staging, and validated questionnaire assessment. The outcomes were evaluated at 6 months, 1 year, and 2 years postoperatively. Examinations were performed by outcome assessors who were not original surgeons and had been trained in the performance of the POP-Q.

All complications were classified via the category-time-site (CTS) system. The CTS system was proposed by the IUGA and ICS in 2011 [13].

### Surgical Technique (Supplementary file 1)

#### Anterior Compartment Mesh Inlay

1. To facilitate dissection, hydrodissection was performed with 80–140 mL of saline with or without adrenaline (Fig. 1b). A midline incision was made in the anterior vaginal wall, extending from the transverse groove of the vagina (bladder neck) to 1 cm distal to the cervical orifice (Fig. 1c).
2. The upper lateral 0.5 cm close to the ischiopubic ramus was chosen as the point of puncture for the distal arm of the mesh. The ischiopubic ramus near the ischial tuberosity was chosen as the puncture point for the proximal arm of the mesh (Fig. 1d).
3. The introducer needles passed through the skin, subcutaneous tissue, thigh fascia lata, adductor muscle tendons, upper pelvic septum, and arcus tendineus fasciae pelvis(ATFP). The mesh was then placed by retracting the introducer needle (Fig. 1e).
4. The distal arm of the mesh was sutured to the anterior wall of the vagina, in front of the middle urethra. The proximal portion of the mesh was sutured to the cervical orifice (Fig. 1f). Before the mesh was fixed, cystoscopy was performed to identify the presence of a bladder, urethra, or ureteral injury or obstruction [14]. The mesh was flattened and sutured on each side via 2-0 absorbable sutures (Fig. 1g).

#### Posterior Colporrhaphy (Rectovaginal Fascial Plication) and Reconstruction of the Perineal Body (Posterior Colpoperineorrhaphy)

1. The reserved vaginal diameter was approximately 3 cm. The reserved diameter of the vagina in elderly women without sexual activity can be properly reduced. A hexagonal incision was made from the outer opening of the posterior vaginal wall to the pelvic diaphragm (Fig. 1h).
2. The posterior vaginal wall was dissected proximal to the cervix and lateral to the pararectal attachments to the pelvic sidewall. The excess vaginal epithelium was trimmed (Fig. 1i). The bilateral rectovaginal fascia, pubococcygeus and puborectalis were then reapproximated to the level of the pelvic diaphragm via a hook-like suture with half-purse-string stitching (Fig. 1j). The upper half of the posterior vaginal epithelium was closed with a 2-0 absorbable suture to the level of the pelvic diaphragm in a locked-stitch fashion (Fig. 1k). The superior and inferior fasciae of the pelvic diaphragm were sutured until the genital hiatus was narrowed.
3. Perineorrhaphy: Suture placement was continued distally and incorporated into the perineal body via three layers. (1) The bilateral ani externus superior and inferior sphincters, superficial and deep transverse perineal muscles, and external anal sphincter subcutaneous layer were continuously sutured by a hook-like suture (Fig. 1l). (2) After dissection of the bilateral external sphincter muscle of the anus at the distal vagina (Fig. 1m), the bilateral external sphincter muscle of the anus at the subvaginal fascia was continuously approximated by a hook-like suture. (3) The lower half of the posterior vaginal epithelium was closed via 2-0 absorbable sutures in a locked stitch fashion (Fig. 1n).
4. The mesh was carefully tightened and cut until the vagina was in a natural state (Fig. 1o).

### Statistical Analysis

Data are presented as the mean ± SD (range). Data that were obtained after wearing a pessary or repeat operations for prolapse recurrence were not included in the analyses, which were performed to eliminate the probability that data would be distorted toward enhanced outcomes after repeat treatments. GraphPad Prism software for Windows (version 6; GraphPad, USA) was used for data management and statistical analysis. Ordinary one-way ANOVA, Tukey’s multiple comparisons test, and χ^2^ tests were used for statistical analysis. Each analysis was structured as a 2-tailed test with α = 0.05.

## Results

The baseline characteristics of the study participants are presented in Table 1. The operative characteristics included a mean blood loss of 54 ± 12 mL (20–100 mL) and a mean operative time of 63 ± 13 min (45–82 min). The mean hospital stay was 4 ± 0.6 days (3–7 days).

**Table 1:** Baseline characteristics of study participants

Characteristics	Participants
Patients, *n*	364
Age, years	60.9 ± 11.7 (32–87)
Postmenopausal	73% (264/364)
Married	100% (364/364)
Body mass index, kg/m^2^	25.1 ± 1.2 (22.4–28.9)
Parity	2.9 ± 1.7 (1–8)
Previous vaginal deliveries	100% (364/364)
Previous hysterectomy	16% (58/364)
Previous surgery for prolapse	2% (8/364)
Hysterectomy	1% (5/364)
Sacrocolpopexy	<1% (2/364)
Sacrospinous ligament fixation	<1% (1/364)
Previous surgery for incontinence	0 (0)
Menopause time, years	12.3 ± 10.5 (0–37)
Chronic obstructive pulmonary disease	29% (104/364)
Hypertension	49% (178/364)
Diabetes mellitus	23% (82/364)
POP-Q stage, *n* (%)	
III	49% (178/364)
IV	51% (186/364)

Data are presented as mean ± SD (range); *N* or % (*n*/*N*). POP-Q: pelvic organ prolapse–quantification

The POP-Q measurements are listed in Table 1. A total of 49% (178/364) and 51% (186/364) of patients had POP-Q stage III and IV deficiencies, respectively, before surgery. Compared with the preoperative POP-Q points, statistically significant improvements in Aa, Ba, C, D, Ap, Bp, Gh, Pb, and TVL were observed at the 6-month, 1-year and 2-year time points (*P* < 0.001). The vaginal and perineal body lengths were longer postoperatively. The genital hiatus was shorter postoperatively (Table 2).

**Table 2:** Anatomical POP-Q stage outcomes and subjective outcomes after physiologic reconstruction of POP

	Preoperation	6-month	1-year	2-year
POP-Q (cm)	*n* = 364	*n* = 356	*n* = 342	*n* = 330
Aa*	2.5 ± 0.9	–2.8 ± 0.6	–2.7 ± 0.8	–2.5 ± 0.7
Ba*	4.2 ± 0.6	–2.6 ± 0.5	–2.5 ± 0.7	–2.4 ± 0.6
C*	–1.1 ± 1.4	–6.3 ± 1.2	–6.1 ± 1.1	–6.0 ± 1.0
D*	–3.6 ± 1.3	–7.2 ± 1.1	–6.9 ± 0.8	–6.8 ± 1.2
Ap*	2.1 ± 1.2	–2.7 ± 0.4	–2.6 ± 0.3	–2.5 ± 0.3
Bp*	3.7 ± 1.3	–2.6 ± 0.4	–2.5 ± 0.4	–2.4 ± 0.3
Gh*	6.1 ± 0.6	3.7 ± 0.4	3.8 ± 0.3	3.8 ± 0.4
Pb*	2.1 ± 0.7	3.4 ± 0.6	3.2 ± 0.5	3.2 ± 0.7
TVL*	5.9 ± 0.8	8.1 ± 0.9	8.0 ± 1.0	7.9 ± 1.0
PFDI-20^a^	144.6 ± 44.5 (36.4, 268.8) (*n* = 364)*	52.1 ± 21.3 (0, 115.6) (*n* = 356)**	13.7 ± 11.1 (0, 62.5) (*n* = 342)	8.1 ± 8.5 (0, 44.7) (*n* = 330)***
Pelvic Organ Prolapse Distress Inventory–6	64.2 ± 18.0 (20.8, 95.8)*	27.2 ± 13.0 (0, 70.8)**	9.2 ± 7.1 (0, 45.8)****	6.1 ± 5.9 (0, 33.3)***
Colorectal Anal Distress Inventory–8	34.6 ± 16.8 (3.1, 90.6)*	10.4 ± 8.9 (0, 65.6)**	1.4 ± 3.0 (0, 22.5)	0.6 ± 2.3 (0, 12.5)***
Urogenital Distress Inventory–6	45.7 ± 23.0 (12.5, 91.7)*	14.4 ± 8.6 (0, 37.5)**	4.0 ± 4.9 (0, 20.8)	2.2 ± 3.7 (0, 16.7)***
PFIQ-7^a^	128.7 ± 35.2 (28.5, 238.2) (*n* = 364)*	50.6 ± 20.4 (0, 128.5) (*n* = 356)**	18.6 ± 11.2 (0, 61.8) (*n* = 342)****	6.4 ± 6.9(0, 33.3) (*n* = 330)***
Pelvic Organ Prolapse Impact Questionnaire–7	61.5 ± 15.6 (14.3, 90.5)*	27.3 ± 12.2 (0, 71.4)**	10.8 ± 6.1 (0, 28.5)****	4.0 ± 4.1 (0, 14.3)***
Colorectal Anal Impact Questionnaire–7	29 ± 14.3 (4.7, 76.2)*	9.3 ± 5.7 (0, 28.5)**	2.5 ± 3.8 (0, 14.3)	0.6 ± 1.9 (0, 9.5)***
Urinary Impact Questionnaire–7	37.8 ± 18.2 (4.7, 81)*	14.0 ± 9.4 (0, 42.9)**	5.5 ± 5.8 (0, 23.8)****	2.1 ± 3.6 (0, 14.3)***
PISQ-12^b^	28.1 ± 4.7 (20.4, 41.5) (*n* = 136)*****	27.9 ± 5.2 (18.2, 42) (*n* = 132)**	31.7 ± 5.4 (18, 43.4) (*n* = 130)	32.6 ± 6.0 (16, 44.6) (*n* = 126)***
Dyspareunia, *n* (%) ^c#^		19% (26/132)	11% (14/130)	5% (6/126)
PGI-I				
Very much better		74% (264/356)	78% (266/342)	80% (264/330)
Much better		22% (78/356)	18% (62/342)	13% (42/330)
A little better		2% (8/356)	2% (6/342)	3% (10/330)
No change		1% (4/356)	1% (4/342)	2% (6/330)
A little worse		<1% (2/356)	<1% (2/342)	1% (2/330)
Much worse		0% (0/356)	0% (0/342)	0% (0/330)
Very much worse		0% (0/356)	0% (0/342)	0% (0/330)
PGI-S				
Normal		69% (246/356)	73% (250/342)	79% (260/330)
Mild		23% (82/356)	21% (72/342)	16% (52/330)
Moderate		8% (28/356)	5% (18/342)	4% (12/330)
Severe		0% (0/178)	1% (4/342)	0% (0/165)

Data are expressed as mean ± SD; *N* or % (*n*/*N*). PFDI-20: Pelvic Floor Distress Inventory-20; PFIQ-7: Pelvic Floor Impact Questionnaire-7; PISQ-12: Pelvic Organ Prolapse/Urinary Incontinence Sexual Questionnaire short form; PGI-I: Patient Global Impression of Improvement; PGI-S: Patient Global Impression of Severity

*Preoperation vs. 6-month, preoperation vs. 1-year, and preoperation vs. 2-year (one-way ANOVA, *P* < 0.001). **6-month vs. 1-year (Tukey's multiple comparisons test, *P* < 0.05). ***6-month vs. 2-year (Tukey's multiple comparisons test, *P* < 0.05). ****1-year vs. 2-year. (Tukey's multiple comparison test, *P* < 0.05). *****preoperation vs. 1-year, preoperation vs. 2-year (Tukey's multiple comparisons test, *P* < 0.05). ^#^6-month vs. 1-year vs. 2-year (χ^2^ test, *P* < 0.05)

^a^ Lower scores represent better outcome; ^b^ higher scores represent better outcome; ^c^ Prolapse and Incontinence Sexual Questionnaire, item 5, response = usually or always

Table 2 lists the subjective outcomes of the PFDI-20, PFIQ-7, PISQ-12, PGI-I, and PGI-S. Statistically significant improvements were observed across all time points for the Pelvic Organ Prolapse Distress Inventory–6, Colorectal Anal Distress Inventory–8, and Urogenital Distress Inventory–6 domains for the PFDI-20 assessment. Significant improvements were also observed in the Pelvic Organ Prolapse Impact Questionnaire–7, Colorectal Anal Impact Questionnaire–7, and Urinary Impact Questionnaire–7 domains on the PFIQ-7 assessment. There were statistically significant differences at 6 months vs. 1 year (*P* < 0.05) and at 6 months vs. 2 years (*P* < 0.05) for the PFDI-20 and PFIQ-7, respectively. A total of 136 patients reported sexual activity at baseline. A total of 126 patients were sexually active at the 2-year follow-up. Statistically significant improvements in the PISQ-12 score were also observed between preoperation and 1 year (*P* < 0.05) and between preoperation and 2 years (*P* < 0.05). Dyspareunia was found in 19% (26/132) of the patients at the 6-month follow-up; this percentage decreased by 11% (14/130) and was 5% (6/126) at the 2-year follow-up. A summary of global impressions of improvement and severity at the 6-month, 1-year, and 2-year follow-ups is listed in Table 2.

Two years after surgery, 15% (50/330) of the patients experienced symptoms of prolapse with a bulge. Among them, 46 patients had POP-Q stage ≤ 1 at 2 years. Only 1% (4/330) of the patients experienced stage II and III recurrence (Table 3). An 87-year-old patient with diabetes mellitus who experienced POP-Q stage IV disease experienced a 3-month follow-up. The repaired perineal body and levator ani muscle did not heal, the vagina was wider, and the vaginal axis was defective again. The patient was satisfied with the use of the vaginal pessary. Another patient relapsed within one month after the operation. The patient experienced constipation before the operation, and the stool was released 7 days after the operation, which led to a long-term increase in abdominal pressure. A physical examination revealed that the patient had a prolonged cervix. The patient recovered after posterior compartment reconstruction and cervical truncation.

**Table 3:** Prolapse by symptoms or anatomy evaluation 2 years after surgery

Variable	2 years after surgery
Prolapse by symptoms (bulge)	15% (50/330)
Recurrent prolapse	1% (4/330)
Reoperation for prolapse	1% (4/330)

Data are % (*n*/*N*)

All complications, according to the CTS system, are presented in Table 4. There were no intra- or postoperative complications of the urethra, bladder, rectum, musculoskeletal, or intra-abdominal complications. Category 1 (4.5%) was due to vaginal epithelium wrinkling or contraction of the vaginal epithelium, and mesh fibers could be palpated in some patients’ vaginal walls. Categories 2 (2.4%) and 3 (1.0%), which refer to mesh exposure, were the most common complications of anterior compartment mesh inlay surgery. The most common complication (2.7%) occurred 2–12 months after synthetic mesh surgery. There were no cases of blood transfusions. Ten percent (36/356) experienced mild thigh or groin pain postoperatively. Fewer than 1% (4/330) of patients required nonsteroidal anti-inflammatory drugs 2 years after the operation. Six percent (22/356) of patients had an overactive bladder postoperatively. Among them, 4% (14/356) had urgent urinary incontinence (Table 5). After treatment with bladder training and the administration of solifenacin succinate, most symptoms were relieved.

**Table 4:** Category-time-site classification of all complications reported

		Number of patients
Total (*n*)		330
Category (*n*, %)	1: Vagina: no epithelial separation	15 (4.5)
	2: Vagina: smaller epithelial separation/exposure/ulcer ≤ 1 cm	8 (2.4)
	3: Vagina: larger epithelial separation/exposure/extrusion > 1 cm	3 (1.0)
	4: Urinary tract	0
	5: Rectal or bowel	0
	6: Skin and/or musculoskeletal	0
	7: Patient compromise	0
Time (*n*, %)	1: <48 h	0
	2: ≥48 h and <2 months	0
	3: ≥2 months and <12 months	9 (2.7)
	4: >12 months	17 (5.2)
Site (*n*, %)	0: No site applicable	0
	1: Vaginal: area of suture line	24 (7.2)
	2: Vaginal: away from area of suture line	2 (0.6)
	3: Trocar passage/adjoining viscus	0
	4: Other skin or musculoskeletal site	0
	5: Intra-abdominal	0

*N* or % (*n*/*N*)

**Table 5:** Complications after physiologic reconstruction of POP

Complication	6-month	1-year	2-year
Thigh or groin mild pain*	10% (36/356)	1% (4/342)	<1% (2/330)
Overactive bladder	6% (22/356)	2% (8/342)	1% (2/330)
Urgent urinary incontinence	4% (14/356)	2% (8/342)	0% (0/330)

Data are expressed as percentages (*n*/*N*). *6-month vs. 1-year vs. 2-year (χ^2^ test, *P* < 0.05)

## Discussion

The key finding of our study was that significant objective and subjective improvements were observed after physiological reconstruction of severe POP. This combined surgical procedure is safe and has a low incidence of associated adverse events.

Younger patient age, concurrent hysterectomy, blood transfusion, and increased patient comorbidity are four risk factors for POP transvaginal mesh complications [15]. The reasons for fewer complications in our study may be older patient age, no concurrent hysterectomy at the time of mesh implantation, no blood transfusion, specialized training and full experience of the surgeon, and ultralight mesh implantation. The findings of this study can be generalized to other fellowship-trained pelvic reconstruction surgeons.

The goal of POP reconstructive surgery is to restore the normal anatomy and function of the pelvic floor organs, including the lower urinary and gastrointestinal tracts and the vagina [16]. Multiple anatomical compartments are often affected by prolapse, with the direction of the pelvic organ vector pointing toward the vaginal orifice. Many studies have reported that the vaginal axis is altered by compensatory prolapse in an untreated compartment. Vaginal mesh surgery for anterior compartment prolapse is more effective than nonmesh surgery but has a greater risk of causing prolapse of other compartments or subsequent incontinence [17]. After vaginal mesh surgery for anterior compartment prolapse, approximately 10% of patients experience mesh erosion [17]. Many transvaginal lasting meshes have been voluntarily withdrawn from the market since 2011, and newer thinner transvaginal lasting meshes have not been assessed in randomized trials.

Synthetic mesh or biological grafts used in the posterior or apical compartments did not improve the success of anatomic or subjective outcomes [18]. Mesh erosion rates ranged from 1.4% to 19% at the anterior vaginal wall, but from 3 to 36% when the mesh was placed in many compartments [18].

Improving the surgical outcomes of POP is a hot topic. The pelvic floor is a three-dimensional structure that functions as a unit. Typically, the pelvic organs depend on three levels of pelvic floor support (pericervical ring, ATFP, pelvic septum, and perineal body). However, vaginal delivery poses the greatest danger to the development of POP later in life. This process can cause laceration of the pericervical ring, ATFP, pelvic septum, or perineal body, which could induce enlargement of the reproductive hiatus, irreversible dilation of the vagina, and loss of the acute vaginal angle [19], and cause the direction of the pelvic organ vector to point to the vaginal orifice.

Therefore, regardless of the type of injury, the entire structure before childbirth should be considered the goal of repair and reconstruction. Physiological repair may yield better therapeutic effects by restoring the normal direction of the pelvic organ vector pointing to the firm sacrococcygeal curve, not the vaginal orifice. Specifically, this combined procedure includes three parts: (A) reconstruction of the first part, repair of the ATFP and the pelvic fascia to reinforce the support of the anterior vaginal wall; (B) reconstruction of the second part, posterior colporrhaphy (rectovaginal fascial plication), which can narrow the width of the vagina and restore the vaginal depth; (C) reconstruction of the third part, perhaps the most important one, (a) repair of the levator ani muscle, which is often found lacerated in POP patients, urinary reproductive diaphragm and pelvic septum to narrow the pelvic septum reproductive hiatus, which is an important independent risk factor for POP [20], and restore the vaginal axis, which could prevent the moment axis of the uterus from facing the vaginal orifice directly; and (b) repair of the injured posterior vaginal wall and rectovaginal fascia during childbirth, restoring the normal diameter and length of the vagina, which could increase the hydrostatic pressure difference between the cervix and vaginal outlet (Supplementary file 2).

In general, the distal trocar used for transvaginal mesh (TVM) surgery is placed approximately 2 cm from the distal aspect of the pubic bone, and the proximal trocar is advanced along the ATFP, exiting 1 cm distal to the ischial spine [21]. We made some modifications to the trocar puncture sites, which were easy to locate. It can also cause less thigh pain because of less injury to the adductor muscles. It can provide a mesh that is firmly anchored to bony structures.

One of the strengths of this study is that we included representative women with POP-Q stage ≥ 3 who would need and most benefit from surgical treatments. We demonstrated that our techniques improve objective and subjective outcomes in this vulnerable population. Additionally, this study was not industry funded and had excellent follow-up evaluations.

The weakness of this research is its retrospective design, which may increase the risk of bias. In future studies, we will perform a proper multicenter, double-blind, prospective RCT with strict inclusion criteria and outcome measures to minimize bias. The lack of a comparison group is another major limitation.

## Conclusions

Restoration of the normal pelvic floor, vaginal morphology, and perineal body is important in women with POP. On the basis of our findings, we demonstrated the benefits of physiological reconstruction of the POP in terms of objective and subjective improvements.

## Supplementary Information

Below is the link to the electronic supplementary material.

Supplementary file1 (MP4 72699 KB)

Supplementary file2 (DOCX 1003 KB)

## Data Availability

Data will be provided upon request to the authors.
