# Supplementary material for: Physiological Reconstruction for Moderate–Severe Pelvic Organ Prolapse: A Multicenter Retrospective Self-Controlled Study
Source: Curr Med Sci. 2025 Jul 30;45(4):909–16. doi: 10.1007/s11596-025-00095-3 (PMC12364984; doi:10.1007/s11596-025-00095-3)
Supplement: Supplementary file 2 — Supplementary file2 (DOCX 1003 KB) [file 11596_2025_95_MOESM2_ESM.docx]

Comparison of the Axes and Positions of the Uterus and Vagina Between Women With and Without Pelvic Floor Organ Prolapse


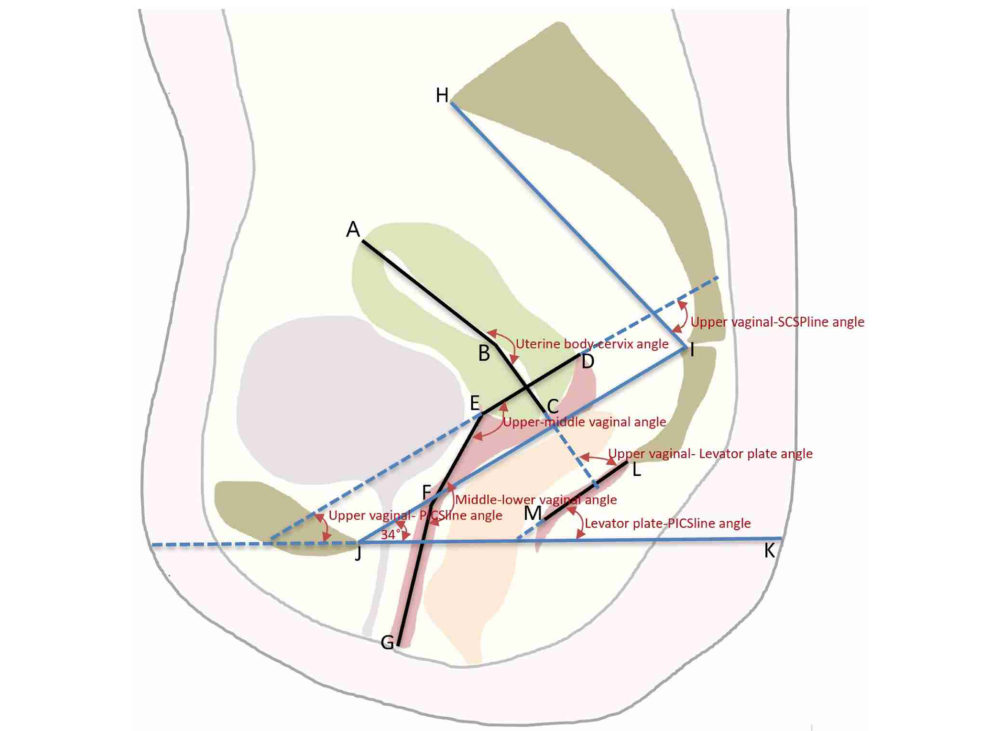


FIGURE 1 | The schematic diagram of the axis and position of the Uterus and Vagina. An illustration of the pelvic landmarks, uterine and vaginal regions, uterine and vaginal axes, and uterine and vaginal angles used in this study is shown. A, uterine floor; B, internal orifice of the cervix; C, external orifice of the cervix; D, posterior vaginal fornix; E, anterior vaginal fornix; F, junction of the middle and lower vagina; G, vaginal introitus; AB, uterine body axis; BC, cervical axis; DE, upper vaginal axis; EF, middle vaginal axis; FG, lower vaginal axis; LM, levator plate; JI, SCIPP sacrococcygeal-inferior pubic point line; JK, pelvic inclination correction system (PICS) line; HI, SCSP line, obtained by connecting a line drawn from the sacral promontory to the junction of the fifth sacral and first coccygeal bone


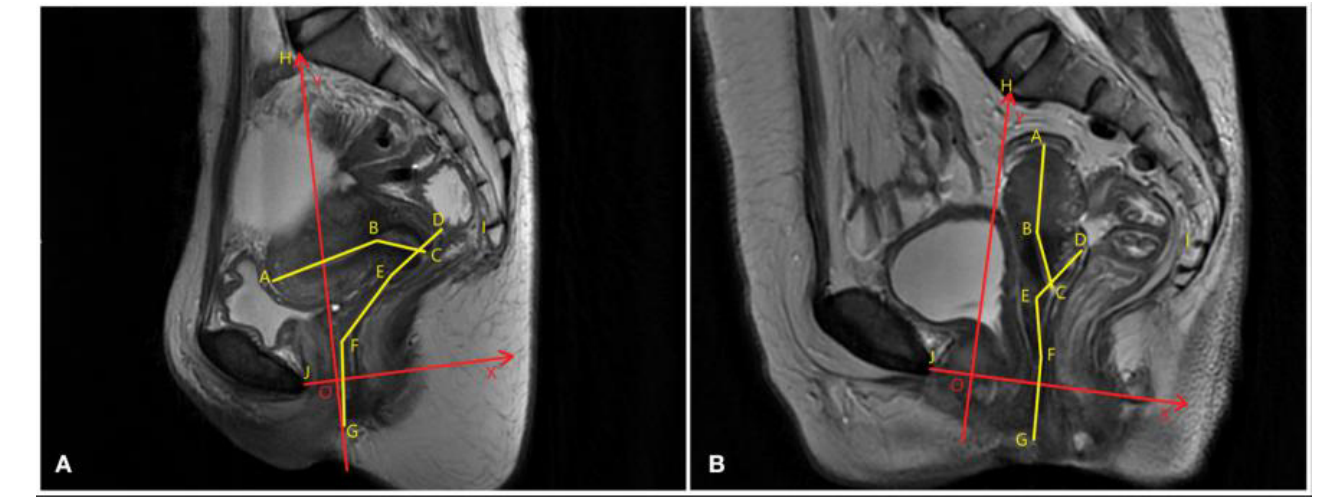


FIGURE 2 | Uterine and vaginal analysis. (A) The central sagittal magnetic resonance (MR) image of a participant without prolapse is shown. (B) The sagittal median MR image of a participant with prolapse is shown. The sagittal plane analysis system of the axes, angles, and positions of uterus and vagina are established. T o compare the shapes, positions, and angles of the uterus and vagina between different participants, a local coordinate system (XOY , in red) that quantifies the morphology was created. The OX axis was created by rotating the sacrococcygeal-inferior pubic point (SCIPP) line 34◦ clockwise. The OY axis is perpendicular to the X-axis through the sacral promontory, and the same coordinate system is used to compare the spatial position of each participant’ s uterus and vaginal axes


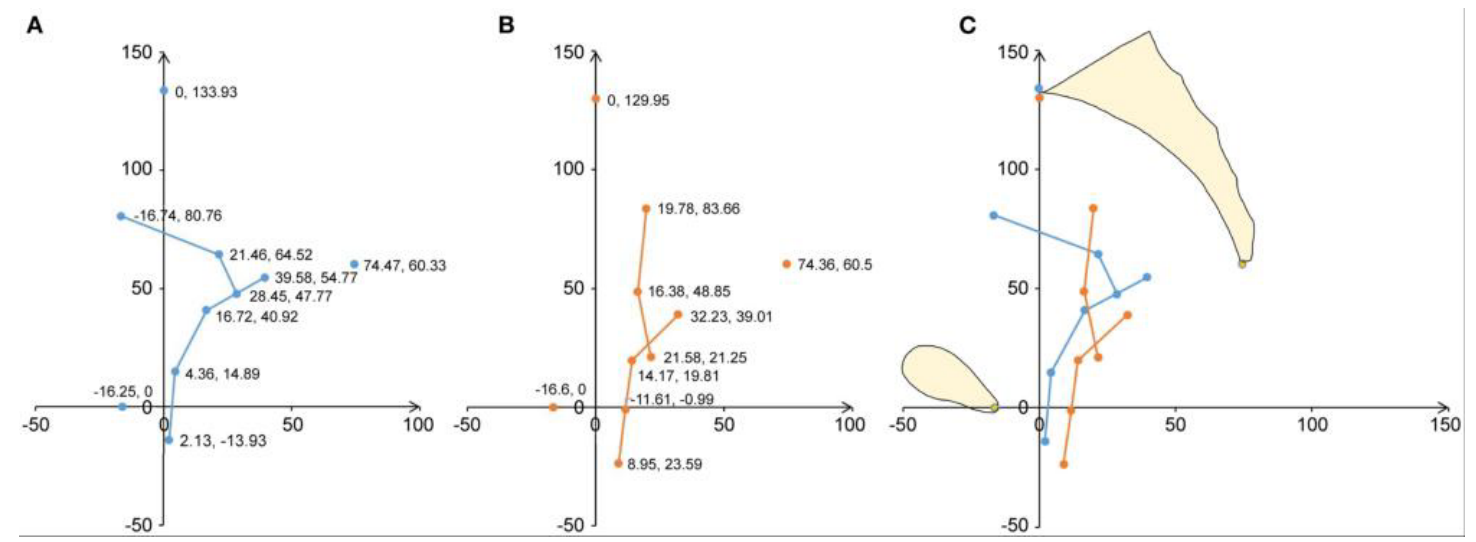


FIGURE 3 | Analysis of the average shape and position of the geometric structure of the uterus and vagina on the median sagittal plane. A mid-sagittal shape and the mean shape of the uterine axis and vaginal axis are shown in terms of the local pelvic inclination correction system (PICS) coordinates. (A) The geometric results of 57 participants without prolapse are shown. The blue dots show the location and coordinate values of the average coordinates of the uterine and vaginal anatomical marks. The blue line indicates the average shape. (B) The geometric results of 81 patients with prolapse are shown. The orange dots show the position and coordinate values of the average coordinates of the uterine and vaginal anatomical marks. The orange line indicates the average shape. (C) The average coordinate positions and shapes of the uterine and vaginal anatomical markers for the prolapse and non-prolapse groups are shown. Orange is the average shape of the prolapse group and blue is the average shape of the non-prolapse group.

Result analysis:

In the prolapse group, the uterine body-cervical angle, cervical-upper vaginal angle, uterine body-PICS line angle, cervical-PICS line angle, and lower vaginal-PICS line angle were smaller and the middle-lower vaginal angle, upper vaginal-PICS line angle, and middle vaginal-PICS line angles were larger than those in the non-prolapse group. The cervical length was longer and the middle and lower vaginal lengths were shorter in the prolapse group. The coordinate system revealed that the uterine and vaginal axes were shifted backward and downward in the prolapse group. Patients in the prolapse group were more likely to have retroversion and retroflexion of the uterus than those in the non-prolapse group. The vagina was shortened, turned forward, and straightened, and the uterus and vagina were shifted backward and downward in the prolapse group. Changes in the axial position of the uterus and vagina are important mechanisms of pelvic floor organ prolapse.
